# [Retracted] PEA15 promotes liver metastasis of colorectal cancer by upregulating the ERK/MAPK signaling pathway

**DOI:** 10.3892/or.2023.8574

**Published:** 2023-05-22

**Authors:** Bo Tang, Wenjin Liang, Yong Liao, Zeming Li, Yan Wang, Chao Yan

Oncol Rep 41: 43–56, 2019; DOI: 10.3892/or.2018.6825

Following the publication of this paper, it was drawn to the Editors' attention by a concerned reader that certain of the tumor images shown in Fig. 4G and H were strikingly similar to tumor images (albeit oriented differently) which had previously appeared in Fig. 8A in another article published in the journal *International Journal of Oncology* [Tang B, Li Y, Yuan S, Tomlinson S and He S: Upregulation of the δ opioid receptor in liver cancer promotes liver cancer progression both *in vitro* and *in vivo*. Int J Oncol 43: 1281-1290, 2013], indicating that results which were purported to have been obtained under different experimental conditions had been derived from the same original source. In view of the fact that these data had already appeared in another publication prior to its submission to *Oncology Reports*, the Editor has decided that this paper should be retracted from the Journal. The authors were asked for an explanation to account for these concerns, but the Editorial Office did not receive a satisfactory reply. The Editor apologizes to the readership for any inconvenience caused.

